# Resveratrol Is Not as Effective as Physical Exercise for Improving Reproductive and Metabolic Functions in Rats with Dihydrotestosterone-Induced Polycystic Ovary Syndrome

**DOI:** 10.1155/2013/964070

**Published:** 2013-04-08

**Authors:** Anna Benrick, Manuel Maliqueo, Sun Miao, Jesus A. Villanueva, Yi Feng, Claes Ohlsson, Antoni J. Duleba, Elisabet Stener-Victorin

**Affiliations:** ^1^Department of Physiology, Institute of Neuroscience and Physiology, The Sahlgrenska Academy at the University of Gothenburg, Box 434, 405 30 Gothenburg, Sweden; ^2^Department of Obstetrics and Gynecology, First Affiliated Hospital, Heilongjiang University of Chinese Medicine, Harbin 150040, China; ^3^Department of Reproductive Medicine, School of Medicine, University of California, San Diego, CA 92103, USA; ^4^Department of Integrative Medicine and Neurobiology, State Key Lab of Medical Neurobiology, Shanghai Medical College, Institute of Acupuncture Research, Institutes of Brain Science, Fudan University, Shanghai 20032, China; ^5^Department of Internal Medicine, Institute of Medicine, Centre for Bone and Arthritis Research, The Sahlgrenska Academy at the University of Gothenburg, 40530 Gothenburg, Sweden

## Abstract

Polycystic ovary syndrome (PCOS) is a reproductive and metabolic disorder associated with obesity and insulin resistance that often precedes the development of type-2 diabetes. Rats continuously exposed to dihydrotestosterone from prepuberty display typical reproductive and metabolic PCOS characteristics including anovulation, polycystic ovaries, insulin resistance, and obesity. Our aim was to investigate if resveratrol improves reproductive and metabolic functions in PCOS rats. The effect was compared to exercise. Control and PCOS rats were treated with vehicle or resveratrol (400 mg *·* kg^−1^ *·* day^−1^) for 5-6 weeks. Another group of PCOS rats received vehicle treatment and exercised for 5-6 weeks. Insulin sensitivity was determined by euglycemic-hyperinsulinemic clamp. The glucose infusion rate was lower in the PCOS-vehicle group compared to control-vehicle rats (*P* < 0.05). Exercise increased insulin sensitivity compared with PCOS-vehicle rats (*P* < 0.05), but resveratrol did not. Resveratrol treatment and exercise resulted in smaller adipocytes, upregulated estrogen-related receptor **α** gene expression in subcutaneous fat, and improved estrus cyclicity in the previously acyclic PCOS rats. Although resveratrol had positive effects on adiposity and cyclicity in a similar manner to exercise, resveratrol does not seem to be a good candidate for treating insulin resistance associated with PCOS because no improvement in insulin sensitivity was observed in PCOS rats on normal chow.

## 1. Background

Polycystic ovary syndrome (PCOS) is a common endocrine and metabolic disorder in women of reproductive age that is associated with obesity and insulin resistance and often precedes the development of type-2 diabetes. Hyperandrogenism is the hallmark of PCOS along with anovulation and polycystic ovaries. Uncontrolled ovarian steroidogenesis, along with a thickened theca cell layer that secretes excessive amounts of androgens, is thought to be the primary abnormality of PCOS [1]. Human studies suggest that excess androgens contribute to many metabolic disturbances [2] and exaggerate sympathetic nerve activity in women with PCOS [3].

PCOS is associated with two strong predictors of insulin resistance: enlarged adipocytes and reduced lipolytic activity [4, 5]. PCOS and type-2 diabetes share common insulin signaling defects in muscle and fat. Insulin resistance in skeletal muscles is associated with abnormalities in fatty acid metabolism and mitochondrial dysfunction, and insulin-stimulated glucose disposal in PCOS patients is associated with reduced gene expression of peroxisome proliferator-activated receptor *γ* coactivator *α* (PGC-1*α*) and sirtuins (SIRT) 1 and 3 [6, 7]. Together, these abnormalities may contribute to the increased risk of type-2 diabetes in PCOS patients.

A rat PCOS model that incorporates both the ovarian and metabolic characteristics of the syndrome can be induced by continuous exposure to dihydrotestosterone (DHT), a nonaromatizable androgen, from prepuberty until adult age [8]. The rats develop a distinct metabolic PCOS phenotype and suffer from obesity that is accompanied by enlarged adipocytes and insulin resistance [8]. They also display typical polycystic ovaries with an increased number of apoptotic follicles but opposite to women with PCOS, these rats have decreased ovarian size [8]. In translational studies, treatment effects of acupuncture on bleeding pattern and decreased circulating testosterone have been successfully predicted using DHT-induced PCOS rats [9–11].

Resveratrol is a natural polyphenol that is found at high concentrations in grapes, berries, nuts, and red wine [12]. A broad range of beneficial metabolic effects have been ascribed to resveratrol, including improvement in insulin sensitivity, a more balanced lipid profile, and decreased adiposity [13]. Furthermore, resveratrol promotes apoptosis and reduces proliferation of rat theca-interstitial cells. It also reduces androgen production primarily by inhibition of Cyp17a1 gene expression [14, 15]. Resveratrol interacts with multiple cellular targets, but many effects of resveratrol are attributed to its activation of SIRT 1 [16], a protein that deacetylates numerous proteins involved in energy and glucose homeostasis. Further, glucose transporter (GLUT)-4 translocation and increased glucose uptake have been reported to be mediated by resveratrol's activation of AMP-activated protein kinase (AMPK) [17]. In turn, AMPK and SIRT 1, have been described to directly act on the downstream PGC-1*α*, a master regulator of mitochondrial biogenesis. However, resveratrol may indirectly activate AMPK, and then SIRT 1, by acting on cAMP signaling [18]. And, although AMPK, SIRT 1, and PGC-1*α* act as a regulatory network for different metabolic needs additional regulators also affect PGC-1*α* activity [19, 20].

Exercise also activates AMPK, and muscle contraction acutely increases glucose transport via GLUT-4 translocation in both healthy and type-2 diabetic individuals in a similar manner as resveratrol. AMPK activation also increases fatty acid oxidation in skeletal muscle by increasing expression of its target genes PGC-1*α* and peroxisome proliferator-activated receptor *α* (PPAR*α*) [21]. Lifestyle intervention studies incorporating increased physical activity with reduced caloric intake show an improvement in ovulatory function, circulating androgen levels, inflammatory pattern, and insulin sensitivity in women with PCOS [22]. Thus, physical exercise and resveratrol both appear to have positive effects on the endocrine and metabolic features of this syndrome. Women with PCOS require long-term treatment and although pharmacological treatments are effective, they have side effects. Thus, there is a need to evaluate new treatment strategies that could regulate reproductive and metabolic functions in women with PCOS.

It is well established that resveratrol exerts beneficial effects on rodents fed a high-calorie diet, but it is not known if giving resveratrol to rats with androgen-induced PCOS can ameliorate the reproductive and metabolic effects of this syndrome. In the current work, we sought to test the hypothesis that resveratrol improves insulin sensitivity in rats on normal chow with DHT-induced PCOS. We also examined the effects of resveratrol on adiposity, fat cell size and lipid profile, bone mineral content, and gene expression related to insulin sensitivity in fat and muscle. In addition, estrus cycle changes and gene expression levels of ovarian enzymes involved in sex steroidogenesis were investigated. The effect of resveratrol was compared to physical exercise, as both appear to have positive effects on PCOS.

## 2. Materials and Methods

### 2.1. Animals

Female Wistar rats were purchased from Charles River (Sulzfeld, Germany) and arrived at 16 days of age with lactating dams. They were fed* ad libitum *with standard rat chow (Harlan Teklad Global Diet, Harlan, Germany) and had free access to water. They were housed under controlled conditions with a 12 : 12-hour light/dark cycle. Animals were cared for according to the principles of the Guide to the Care and Use of Experimental Animals (http://www.sjv.se/). The animal Ethics Committee at the University of Gothenburg approved the study.

### 2.2. Study Procedure and Treatment

At 21 days of age, female Wistar rats were separated from the lactating dam and implanted subcutaneously with a slow-releasing DHT pellet (Innovative Research of America, Sarasota, FL) containing 7.5 mg DHT (daily dose, 83 *μ*g) to induce PCOS. Control rats received a placebo pellet. The pellets were inserted under light anesthesia with isoflurane (Isoba vet, Schering-Plough AB, Stockholm, Sweden) together with a microchip (AVID, Norco, CA) with an identification number. At 9 weeks of age, the control rats were subdivided into the control-vehicle and control-resveratrol control groups and the PCOS rats were subdivided into the PCOS-vehicle, PCOS-resveratrol, and PCOS-exercise experimental groups (*n* = 10/group). The resveratrol groups were given 400 mg · kg^−1^ · day^−1^ resveratrol (Orchid Pharma, Chennai, India) in 0.7% carboxymethylcellulose (CMC) in PBS (Sigma-Aldrich, MO, USA), and all other groups were given 0.7% CMC vehicle solution [13, 23]. The suspension was freshly prepared and shaken vigorously before oral gavage. Resveratrol or vehicle was administrated 5 days a week for 4 weeks and for all 7 days of the final treatment week.

**Figure 1:**
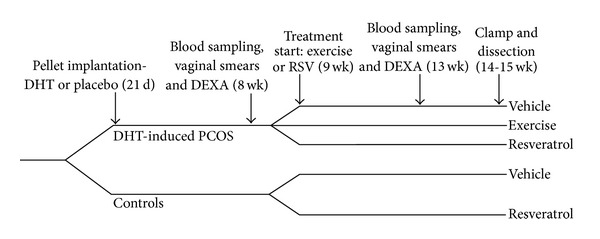
Outline of study design. DHT: dihydrotestosterone; d: days of age; wk: weeks of age; DEXA: dual-energy X-ray absorptiometry; RSV: resveratrol.

Rats in the exercise group were housed singly and had free access to a running wheel (0.34 m in diameter). They were allowed to exercise voluntarily for 5 weeks, and customized computer software registered all wheel rotations. The wheels were locked 24 h prior to performing the clamp studies. Body composition measurements (DEXA), vaginal smears, and blood samplings were taken before and after 5 weeks of treatment, at 8 and 13 weeks of age.  Figure 1 shows the outline of the study design.

### 2.3. Vaginal Smears and Blood Sampling

The estrus cycle stage was determined by microscopic analysis of the predominant cell types obtained via the vaginal smears taken daily [24] when the rats were 8 and 13 weeks of age (Figure 1). Tail blood samples for determining the lipid profile were obtained after a 5 h fast, at the same time as the vaginal smears. Blood samples were obtained in the estrus phase in control rats, and samples were taken from the PCOS rats regardless of estrus phase because these rats were acyclic and displayed a chronic pseudo diestrus [8, 9]. The samples were immediately centrifuged and plasma and serum were stored at −80°C.

### 2.4. Body Composition and Bone Density Measurements

Body composition and bone density were analyzed by dual-emission X-ray absorptiometry (DEXA, QDR-1000/W; Hologic, Waltham, MA) at 8 and 13 weeks of age. The rats were anesthetized by inhalation of isoflurane (Isoba vet, Schering-Plough AB, Stockholm, Sweden) during the scanning procedure.

Bone density was also analyzed *ex vivo* by peripheral quantitative computerized tomography (pQCT). CT scans were performed with a Stratec pQCT XCT Research M (v5.4B; Norland-Stratec, Fort Atkinson, WI, USA) operating at a resolution of 70 *μ*m. Trabecular volumetric bone mineral density (BMD) was determined with a metaphyseal pQCT scan of the distal femur and was defined as the inner 45% of the total area. Bone lengths were measured with a slide caliper. Cortical volumetric BMD was analyzed in the mid-diaphyseal region of the femur.

### 2.5. Euglycemic-Hyperinsulinemic Clamp

The euglycemic-hyperinsulinemic clamp was performed in the estrus phase in controls and in cyclic PCOS rats, and regardless of cycle day in acyclic PCOS rats. After 5 weeks of treatment, at 14-15 weeks of age, the rats were subjected to a euglycemic-hyperinsulinemic clamp as previously described [8]. Briefly, rats were anesthetized with thiobutabarbital sodium (Inactin; Sigma, St. Louis, MO). Insulin (Actrapid; Novo Nordisk, Bagsvaerd, Denmark) diluted in 10 mL saline plus 0.2 mL albumin was infused at a rate of 8 mU · min^−1^ · kg^−1^. Plasma glucose levels were analyzed every 5 min with a OneTouch Ultra 2 Meter (LifeScan, Inc., Milpitas, CA, USA) and were maintained between 5.9 and 6.2 mM by the administration of 20% glucose in saline. At baseline and at steady-state blood samples were taken to determine plasma insulin concentrations. The mean glucose infusion rate (GIR) was normalized to body weight.

### 2.6. Computerized Determination of Adipocyte Size

Inguinal and mesenteric adipose tissues were cut into small pieces and treated with collagenase (Type A; Roche, Mannheim, Germany) in minimum essential medium (1.5 mg/mL; Invitrogen, Carlsbad, CA) containing 5.5 mM glucose, 25 mM HEPES, 4% bovine albumin (Fraction V), and 1.5 *μ*M adenosine (pH 7.4) for 50 min at 37°C in a shaking bath. The samples were washed three times and suspended in fresh medium after filtration through a 250 *μ*m nylon mesh. The cell suspension was placed on a siliconized glass slide with a coverslip. Twelve random visual fields were photographed with a CCD camera (Axiocam, Carl Zeiss), and the mean cell size and size distribution were determined by computerized image analysis using Leica software (Leica Qwin V3, Leica microsystem) [25]. Uniform microspheres 98 *μ*m in diameter (Dynal, Invitrogen Corporation, Oslo, Norway) served as a reference.

### 2.7. Analytical Methods

The levels of human insulin, administered during the clamp, and rat insulin were measured with ELISA kits (10-1113-01 and 10–1251-01, resp.; Mercodia, Uppsala, Sweden). The intra-assay and interassay coefficients of variation and sensitivity were 3.4% and 3.0%, respectively, for human insulin and 2.0% and 4.2%, respectively, for rat insulin. The homeostasis model assessment (HOMA) index was calculated using the formula (fasting blood glucose (mmol/L)) × (fasting insulin (mU/L))/22.5. Serum lipid profiles were determined enzymatically on a Konelab 20 autoanalyzer (Thermo Fisher Scientific). The interassay coefficients of variation were <3%.

### 2.8. RNA Preparation and RT-PCR

Ovary RNA isolation and the reverse transcription of ovarian RNA to cDNA was performed according to Ortega et al. [26]. Quantitative real-time PCR reactions were run on a 7300 Real-Time PCR instrument (Applied Biosystems) and were performed in triplicate. Known concentrations of cDNA were included to generate standard curves. SYBR green was used to detect the rat-specific primer sequences shown in Table 1. Melting curves were performed to verify the PCR products. RNA isolation of tibialis muscle and inguinal fat was done using commercial kits (74704 and 74804, resp.; Qiagen, Germany). cDNA was prepared using SuperScript VILO (Life technologies, Paisley, UK) according to the manufacturer's protocol. Quantitative real-time PCR reactions were performed in duplicate using TaqMan assays (Applied Biosystems) (Table 1) and the 7900HT Fast Real-Time PCR System (Applied Biosystems). Data were analyzed using SDS 1.4 software (Applied Biosystems). Of four putative reference genes (Table 1), hypoxanthine phosphoribosyl transferase (*Hprt*) was chosen as the reference gene for muscle and ovary tissues, and peptidylprolyl isomerase A (*Ppia*) was chosen for fat tissue. Gene expression values were calculated using the 2^−ΔΔCt^ method.

### 2.9. Statistical Analyses

All statistical analyses were performed with SPSS software (version 19.0; SPSS, Inc., Chicago, IL). Effects on body weight were analyzed by repeated measures ANOVA, and DEXA and pQCT measurements were analyzed by one-way ANOVA with Dunnett's *post hoc* test with the PCOS-vehicle group serving as the control category. Estrus cyclicity was analyzed using the Chi-square test. For all other variables, comparisons between the PCOS-vehicle and control-vehicle groups used the Mann-Whitney *U* test to confirm phenotypes. The Mann-Whitney *U* test was also used for comparisons between the control-vehicle and control-resveratrol groups, the PCOS-vehicle and PCOS-resveratrol groups, and the PCOS-vehicle and PCOS-exercise groups. The correlation analysis between the GIR and adipocyte size was performed using the bivariate Spearman rank correlation coefficient (*R*_s_). Values are reported as mean ± SEM, and *P* ≤ 0.05 was considered significant.

## 3. Results

### 3.1. Physical Exercise

The PCOS-exercise group reached maximal running activity after 4 weeks of free access to running wheels. On average, the rats ran 1.7 ± 0.3 km/day during week 1, 2.3 ± 0.4 km/day during week 2, 5.2 ± 1.2 km/day during week 3, 5.9 ± 1.2 km/day during week 4, and 4.8 ± 1.2 km/day during week 5.

### 3.2. Body Weight and Serum Triglycerides Increase in PCOS Rats

**Figure 2:**
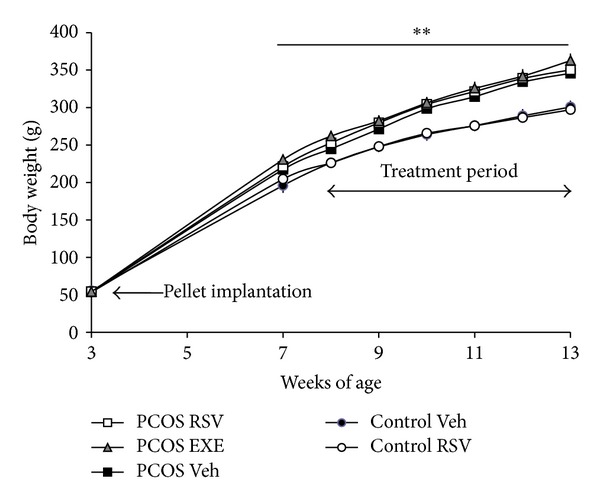
Body weight curves. Rats were exposed to placebo or DHT-containing pellets from 21 days of age until the end of the experiment. The exercise and resveratrol treatments started at 9 weeks of age. Values are presented as mean ± SEM. Statistics were calculated using repeated measurement ANOVA with Dunnett's *post hoc* test. **P < 0.01 control-vehicle versus PCOS-vehicle. EXE: exercise; RSV: resveratrol; Veh: vehicle.

After 4 weeks of DHT exposure and onwards, the PCOS groups gained weight compared with the control groups (Figure 2). No differences in body weight were seen between the PCOS-resveratrol and PCOS-exercise groups compared with the PCOS-vehicle group. The exercise group had a slower rate of weight gain during the first two weeks of exercise, but there was no difference in body weight at the end of the study. Food intake was higher in the exercise group than in all other groups (29.0 ± 0.5 g versus 23.4 ± 0.7 g, *P* < 0.001), and there was no change in food intake between vehicle- and resveratrol-treated controls and PCOS rats (data not shown).

  Serum triglyceride levels were higher in PCOS rats than in controls but did not change during treatment (Table 2). High-density lipoprotein (HDL) and cholesterol levels were lower in the control-resveratrol group compared with the control-vehicle group, but no exercise- or resveratrol-induced changes in lipid profile were seen in PCOS rats. Serum concentrations of low-density lipoprotein (LDL) were similar in all groups (Table 2).

### 3.3. Physical Exercise, but Not Resveratrol, Improves Insulin Sensitivity

**Figure 3:**
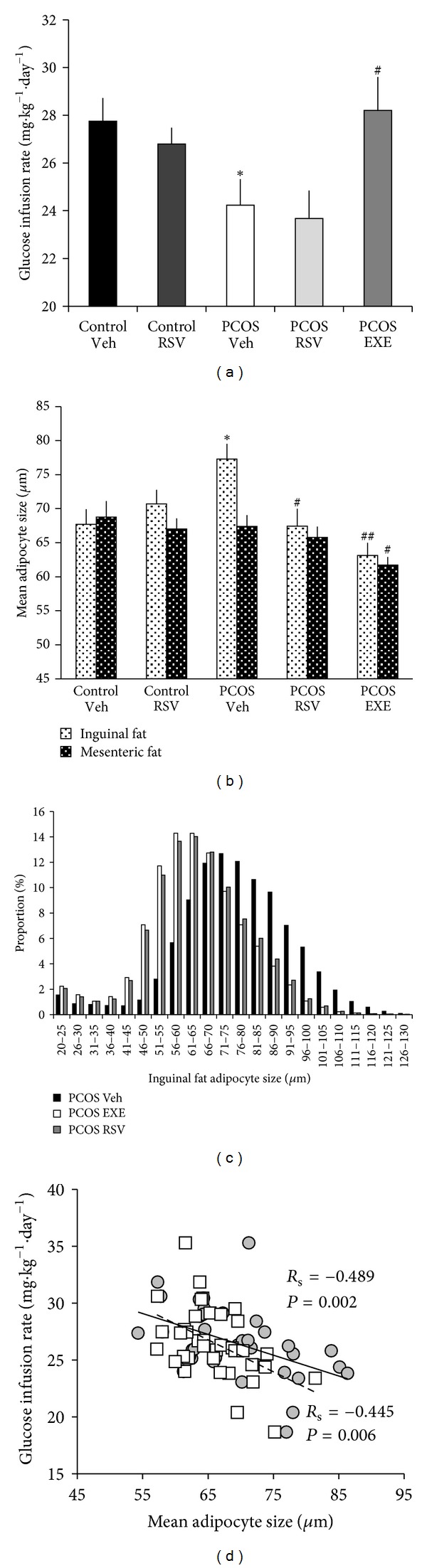
(a) Glucose infusion rate during hyperinsulinemic euglycemic clamp, (b) mean adipocyte size, (c) fat cell size distribution curves, and (d) correlation between fat cell size and glucose infusion rate in mesenteric (squares) and inguinal (circles) adipocytes. Statistics were calculated using the bivariate Spearman rank correlation coefficient (*R*_s_) for correlation analysis and the Mann-Whitney *U* test for group comparisons. *P < 0.05 versus control-vehicle, ^#^ *P* < 0.05, ^##^ *P* < 0.01 versus PCOS-vehicle. EXE: exercise; RSV: resveratrol; Veh: vehicle.

The GIR was lower in the PCOS-vehicle group than in controls, which indicates decreased insulin sensitivity (Figure 3(a)), and the PCOS-exercise group had a higher GIR compared to the PCOS-vehicle group (Figure 3(a)). At steady-state, the plasma glucose level was approximately 5.9–6.2 mmol/L, and the plasma insulin levels were 232.4 ± 9.1 mU/L (control-vehicle) and 279.2 ± 18.7 mU/L (PCOS-vehicle, n.s.). No significant difference in the GIR was found between the control-resveratrol and control-vehicle groups or between the PCOS-resveratrol and PCOS-vehicle groups (Figure 3(a)). Fasting blood glucose levels, fasting insulin levels, and calculated HOMA indices did not differ between groups after treatment (Table 2).

### 3.4. Exercise and Resveratrol Treatments Decrease Adiposity

DEXA measurements were performed at 8 and 13 weeks of age to determine the body composition before and after treatment. At 5 weeks after pellet implantation, PCOS rats had more total body fat (32.9 ± 1.2 g versus 26.4 ± 1.4 g, *P* < 0.01) and total lean mass (192.3 ± 3.2 g versus 172.3 ± 3.4 g, *P* = 0.001), but not in relation to body weight (data not shown), compared with controls. The differences in fat and lean mass weight gain between PCOS-vehicle and control-vehicle animals persisted until 13 weeks of age (Table 3). The PCOS-exercise group had less body fat and more lean mass compared to PCOS-vehicle rats. The fat mass gain was lower in the PCOS-resveratrol group than in the PCOS-vehicle group after treatment (Table 3).

The weights of the total dissected fat (14.1 ± 0.9 g versus 11.1 ± 0.9 g, *P* < 0.05) and inguinal adipose tissue were higher in the PCOS-vehicle group than in the control-vehicle group (Table 4). The PCOS-vehicle group also had more inguinal and mesenteric fat in relation to body weight than the control animals (Table 4). The PCOS-exercise group had a reduced amount of total dissected fat (10.2 ± 0.9 g versus 14.1 ± 0.9 g, *P* < 0.01) as well as inguinal, parametrial, and retroperitoneal fat depots compared to PCOS-vehicle rats (Table 4). There was no effect of resveratrol treatment on dissected fat content compared to respective control groups.

### 3.5. Resveratrol and Exercise Decrease Inguinal Adipocyte Size

Adipocyte size and distribution were determined in inguinal and mesenteric fat tissues. In PCOS-vehicle rats, the mean inguinal adipocyte size was larger than in controls (Figure 3(b)), and the size distribution was shifted to the right (data not shown). Both exercise and resveratrol treatment in PCOS rats led to decreased mean inguinal adipocyte size and shifted the size distribution to the left (Figure 3(c)). In mesenteric fat, mean adipocyte size (Figure 3(b)) and the size distribution (data not shown) did not differ between PCOS-vehicle and control animals. Only exercise had an effect on mesenteric adipocytes, which were significantly smaller compared to control and PCOS-vehicle rats (Figure 3(b)). In each cell population, between 636 and 5514 cells (mean 2725 ± 132) were analyzed, and the mean size of the analyzed reference microspheres was 97.89 ± 0.49 mm.

Insulin sensitivity correlated negatively with inguinal and mesenteric adipocyte size when all animals were included (Figure 3(d), *n* = 37). Only inguinal adipocyte size correlated with the GIR in pooled control rats (Rs = −0.729, *P* < 0.01, *n* = 15), but both inguinal (Rs = −0.452, *P* < 0.05, *n* = 22) and mesenteric (Rs = −0.487, *P* < 0.05, *n* = 22) adipocyte size correlated with the GIR in pooled PCOS rats.

### 3.6. Exercise and Resveratrol Treatment Improve Estrus Cyclicity in PCOS Rats

**Figure 4:**
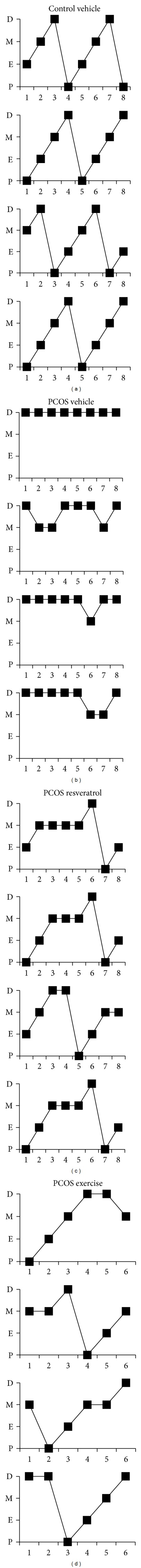
Estrus cycle patterns after 4-5 weeks of treatment (13 weeks of age) in four representative rats from each group except control-RSV, which also had a normal estrus cycle like control-vehicle. D: diestrus; E: estrus; M: metestrus; P: proestrus.

All rats in the control-vehicle (Figure 4(a)) and control-RSV (data not shown) groups had a normal estrus cycle of 4 days, and the rats in the PCOS vehicle group were acyclic (Figure 4(a)). Vaginal smears of all PCOS rats before treatment showed leukocytes, the dominant cell type of the diestrus phase, indicating a chronic pseudo diestrus phase in these rats. After 4 weeks of voluntary exercise or resveratrol treatment, PCOS rats showed changes in estrus cycle in which 4 or 5 animals per group were in estrus phase at least once during a 6-day period, but only one animal in the PCOS-vehicle group was in estrus during the same period (Figures 4(c) and 4(d), *P* < 0.01 and *P* < 0.001 for the PCOS-resveratrol and PCOS-exercise groups, resp.). Ovary and uterus weights were lower in PCOS rats than in controls (Table 4), and there was a tendency for the ovaries in the PCOS-exercise group to be larger compared to PCOS-vehicle rats (*P* = 0.066).

We measured the ovarian gene expression of three enzymes involved in sex steroidogenesis (3-beta-hydroxysteroid dehydrogenase (3*β*-HSD*/Hsd3b1*), 17*α*-hydroxylase (*Cyp17a1*), and 11*α*-hydroxylase (*Cyp11a1*)) by RT-PCR. The control-RSV group was excluded from the gene expression analysis since we did not detect any significant metabolic or reproductive changes in this group compared to the control-vehicle group.* Cyp17a1* was upregulated and *Cyp11a1* was downregulated in PCOS-vehicle rats compared to vehicle controls, but neither exercise nor resveratrol treatment had any affect on the gene expression levels (Table 5). In the PCOS-exercise group, *Hsd3b1* expression was significantly higher compared to PCOS-vehicle rats (Table 5). When the data were divided into responders and nonresponders based on ovulation pattern (anovulatory PCOS rats versus PCOS rats that exhibited an estrus cycle changes), *Cyp11a1 *gene expression levels were higher in the PCOS-exercise and PCOS-resveratrol rats that responded to the treatment than in the PCOS-vehicle rats (Table 5). The *Cyp17a1* gene expression levels did not differ between responders and nonresponders compared to PCOS-vehicle rats (Table 5). The *Hsd3b1* expression levels were significantly higher in responding exercise rats, but *Hsd3b1* mRNA levels did not differ in nonresponders compared to PCOS-vehicle animals (Table 5).

### 3.7. Estrogen-Related Receptor *α* Levels Increase with Resveratrol and Exercise

**Figure 5:**
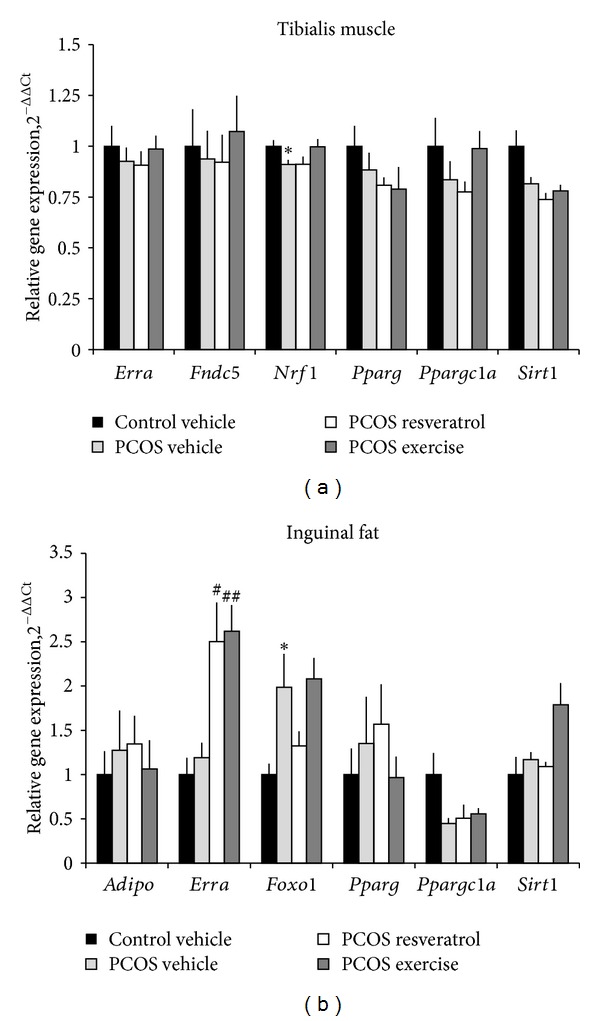
Gene expression in (a) tibialis muscle and (b) inguinal fat. The data are expressed as relative gene expression shown as fold change over control group and presented as mean ± SEM of the ΔΔCT values. Statistics were calculated using the Mann-Whitney *U* test. *P < 0.05 versus control-vehicle and ^#^ *P* < 0.05, and ^##^ *P* < 0.01 versus PCOS-vehicle. *Adipo*: adiponectin; *Erra*: estrogen-related receptor alpha; *Fndc5*: irisin; *Foxo1*: forkhead box O; *Nrf1*: nuclear respiratory factor 1; *Pparg*: peroxisome proliferator-activated receptor gamma; *Ppargc1a*-peroxisome proliferator-activated receptor gamma coactivator 1 alpha; *Sirt1*: sirtuin 1.

In tibialis skeletal muscle, gene expression of nuclear respiratory factor 1 (*Nrf1*) was lower in PCOS-vehicle rats than in control-vehicle rats (Figure 5(a)). Exercise tended to increase mRNA expression of* Nrf1* (*P* = 0.06) but had no effect on any of the other skeletal muscle genes that were studied in this work (Figure 5(a)). In the inguinal fat, expression of the forkhead box O1 (*Foxo1*) gene was up-regulated in PCOS-vehicle rats compared to control-vehicle rats. Inguinal expression of the estrogen-related receptor *α* (*Erra*) gene also increased in both PCOS-exercise and PCOS-resveratrol rats compared to PCOS-vehicle rats (Figure 5(b)).

### 3.8. Bone Density Decreases in PCOS Rats

Bone composition, measured *in vivo *by DEXA and *ex vivo* by pQCT, was lower in PCOS-vehicle rats than in control-vehicle rats. At the start of treatment at 8 weeks of age, PCOS rats had lower BMD (120.2 ± 0.3 versus 125.2 ± 0.9 mg/cm^2^, *P* < 0.001) and lower bone mineral content (BMC) in relation to body weight than control rats (Table 3). Total BMC was increased in PCOS rats compared with control rats (5.67 ± 0.09 versus 5.36 ± 0.08 g, *P* < 0.05). The lower BMD was maintained because total BMD and trabecular BMD as measured by pQCT were lower in PCOS-vehicle rats than in control-vehicle rats after treatment (Table 3). Neither exercise nor resveratrol treatment could reverse the bone loss seen in PCOS animals (Table 3). Cortical BMD was not affected by DHT exposure or by exercise or resveratrol treatment (Table 3).

## 4. Discussion

### 4.1. Exercise, but Not Resveratrol, Restores Altered Insulin Sensitivity

The major finding in the present study is that although resveratrol had effects on both reproductive and metabolic parameters, it did not restore insulin sensitivity in rats with androgen-induced insulin resistance. In diabetic and diet-induced obese animal models, resveratrol treatment has been shown to lower blood glucose levels and improve insulin sensitivity [27–33]. Many previous resveratrol studies used diabetic or high-fat diet-induced insulin-resistant animal models [27–29]. The DHT-induced rat PCOS model used in the current study is associated with androgen-induced obesity and reduced insulin sensitivity even when maintained on a normal chow diet [8, 34]. Therefore, the absence of improvement in insulin sensitivity in this study might be due to mechanisms of insulin resistance in PCOS that are distinct from those in type-2 diabetes. Moreover, many studies start with resveratrol treatment and high-fat diet simultaneously, suggesting a preventive rather than curative effect of resveratrol on insulin resistance. However, we did not see any improvement in glycemic control of any metabolic indicators such as the GIR or the fasting blood glucose and insulin levels after resveratrol treatment. The lack of a high-fat diet might also explain why there was no significant difference in body weight between the vehicle and resveratrol groups after treatment in both the control and PCOS rats. Furthermore, resveratrol supplementation in non-obese women with normal glucose tolerance [35] and in control animals [28, 36] did not have any beneficial metabolic effects. This is in line with our data in which we did not see any *in vivo* effects of resveratrol treatment in metabolically healthy control rats.

There is a large body of evidence from clinical and experimental studies demonstrating that exercise improves or restores insulin resistance in diabetic and obese subjects [21, 32, 33] and in women with PCOS [37]. This confirms our previous findings showing that exercise also restores insulin sensitivity in PCOS rats [34]. The effects on fat mass and adipocyte size and the long-lasting effects of muscle contractions to stimulate insulin-independent glucose uptake most likely explain the effects of exercise on insulin sensitivity in PCOS rats [21, 34]. 

### 4.2. Exercise and Resveratrol Decrease Adiposity

Although resveratrol did not have an effect on insulin sensitivity and body weight, the resveratrol-treated PCOS rats showed decreased fat mass gain and smaller adipocytes. The exercise group also displayed reduced adiposity and adipocyte size, in line with previous studies showing that exercise-induced weight loss efficiently reduces adipocyte size [34, 38]. The effect on fat mass was not because of decreased food intake because the amount of food consumed was unchanged. In regards to resveratrol, similar results were found in rats on a high-fat diet in which treatment had no effect on body weight or food intake but did lead to decreased adipose tissue weight [39]. However, resveratrol-treated mice on a high-fat diet had both decreased body weight and fat pad weight [30, 40]. Because resveratrol decreases lipogenic enzyme activity and stimulates adipose triglyceride lipase, the body fat lowering effect of resveratrol may be mediated by the induction of lipolysis and the reduction of lipogenesis and fatty acid uptake [39, 41]. 

Resveratrol may also alter fat mass by directly affecting adipogenesis by inhibiting adipocyte proliferation and differentiation and inducing apoptosis [42]. Although the mechanism responsible for this has yet to be determined, we found that resveratrol treatment reduced inguinal adipocyte size while exercise reduced both mesenteric and inguinal adipocyte cell size. It is likely that a combination of reduced lipid uptake and storage together with decreased adipogenesis contributes to the reduced adipocyte size.

Exercise is proven to be effective in improving lipid profiles [32], but resveratrol has only shown beneficial changes in some [28, 30], but not all, studies on rodents with diet-induced obesity [27, 31, 40, 43]. On normal chow, serum triglyceride levels were higher in PCOS rats than in controls, and neither exercise nor resveratrol affected plasma levels. 

### 4.3. Muscle and Adipose Tissue Gene Expression

Inguinal expression of *Erra*, which mediates many of the downstream effects that activated PGC-1*α* has on mitochondrial function, was markedly increased by resveratrol treatment and exercise, suggesting increased energy needs in these animals [44]. We have not measured circulating sex hormones in this study, but these may change with exercise as has been demonstrated in women with PCOS [10] and by resveratrol treatment [14]. Circulating sex hormones could possibly affect inguinal fat *Erra *gene mRNA expression because this gene is regulated by estrogen and progesterone *in vitro *[45].

In skeletal muscle, resveratrol has been shown to increase the mRNA levels of *Erra*, *Ppargc1a*, and the ERR*α*/PGC-1*α* signaling pathway target *Nrf1* [31, 40]. However, in the present study, the expression of these genes did not change upon resveratrol treatment while exercise tended to restore *Nrf1 *expression in the soleus muscle. The discrepancy between our study and other studies [31, 40] may, again, be the lack of a high-fat diet in our study. Furthermore, mRNA levels of *Nrf1* were significantly decreased in skeletal muscle in PCOS animals. This is in line with clinical data showing reduced levels of *SIRT1* and *PPARGC1A* in the skeletal muscles of women with PCOS [6, 7]. 

### 4.4. Neither Exercise Nor Resveratrol Has an Effect on Bone Mineral Density

Although our PCOS rats already had a significant decrease in BMD after 5 weeks of DHT exposure, neither exercise nor resveratrol treatment had any effect on total, trabecular, or cortical BMD. Exercise training is important for the maintenance of bone mass in humans and can increase bone mass in rodents, and weight-bearing loading has been found to be more effective than endurance exercise [46]. Endurance exercise, however, has been shown to increase trabecular bone mass in ovariectomized rats but not in intact animals [47, 48].

Resveratrol acts by antagonizing osteoclast and promoting osteoblast differentiation *in vitro* [49, 50]. The osteogenic effects *in vivo* are limited, but resveratrol has been shown to improve BMD in ovariectomized rodents [51, 52] and in normal mice with age-induced bone loss [53] indicating that resveratrol may improve bone health. These animals were treated with resveratrol for 2.5–6 months, and the resveratrol treatment started at the same time as the bone loss [51–53]. It is possible that the treatment period of 5-6 weeks in the current study was not enough to promote osteoblast differentiation and bone formation and that the bone loss had already been established at the start of treatment.

### 4.5. Exercise and Resveratrol Improve Estrus Cyclicity and Steroidogenesis

An improvement in menstrual cyclicity was found in both the PCOS-resveratrol and exercise groups in which 4-5 rats/group were in estrus phase at least once during a 6-day period, although it must be noted that they did not have regular cycles. One possible explanation for the improved cyclicity is that decreased sympathetic activity will have a direct impact on the ovaries and affect the sex steroid synthesis pathways. It has been shown that physical exercise can decrease sympathetic nerve activity, improve menstrual frequency, and decrease the levels of several sex steroids in women with PCOS [10, 54]. In DHT-induced PCOS rats, exercise improves estrus cyclicity and downregulates sympathetic markers, for example, nerve growth factor and neuropeptide Y [9]. The improvement in cyclicity was seen in both groups, but it is not known if resveratrol affects sympathetic activity in a similar manner as physical exercise. Another explanation is that reduced fat mass subsequently lowers leptin levels and improves cyclicity. High leptin levels inhibit ovulation in rats [55] and both exercise and resveratrol decrease leptin levels [28, 34, 56]. 

 Resveratrol promotes apoptosis and reduces rat theca-interstitial cell growth *in vitro *[15]. Theca cells exposed to resveratrol also show reduced androgen production and mRNA expression of *Cyp17a1*, a gene that regulates androgen production [14]. *Cyp17a1* expression was higher in all PCOS groups and was not affected by either exercise or resveratrol treatment suggesting that the DHT exposure further enhanced androgen production. *Cyp11a1*, located in the mitochondria, was downregulated in PCOS rats and tended to be increased by exercise although it did not reach a level of significance (*P* = 0.058). This is in line with the hypothesis that mitochondrial function is impaired in PCOS [6, 7] and enhanced by exercise [21, 32]. However, when the data were divided into responders and nonresponders based on ovulation pattern it was clear that *Cyp11a1* levels were restored in the responding exercise- and resveratrol-treated PCOS rats. A sevenfold increase in *Cyp17a1* mRNA expression was seen in nonresponding rats compared to controls. There was no significant decrease in ovarian *Cyp17a1 *gene expression in the responding resveratrol-treated PCOS rats as shown *in vitro* in theca cells [14]. This is likely due to the low number of animals used and it would be interesting to study the *in vivo* effect of resveratrol on ovarian steroidogenesis and fertility in a larger number of animals.

The exercise group had the highest number of rats in the estrus phase, and *Hsd3b1 *expression was elevated. Some of the beneficial effects of exercise may be mediated through the metabolism of hormonal steroids because 3*β*-HSD is essential for the biosynthesis of progesterone, androgens, and estrogens. Exercise may improve circulating sex hormones in a similar manner as electrical stimulation acupuncture, evoking muscle twitches, without affecting exogenous DHT levels [11]. This does not, however, seem to be the case for resveratrol treatment because *Hsd3b1* expression was unaffected by resveratrol treatment in both responders and nonresponders.

### 4.6. Dose Selection of Resveratrol

The dose of 400 mg/kg was based on previous studies in which doses ranging from 10 to 400 mg/kg displayed positive effects on adiposity and insulin sensitivity [13]. Very few adverse effects have been documented due to the low bioavailability of resveratrol and no side effects were seen in this study. Attempts to increase the bioavailability of resveratrol have improved the rate at which resveratrol is absorbed but not the total amount absorbed [23]. Therefore, resveratrol was dissolved in CMC in this study because it has been shown to have as good oral bioavailability as other tested formulations [23]. Resveratrol had effects, but was not as effective as exercise on several end points, indicating that it was bioactive. We have not measured plasma concentrations of resveratrol because plasma levels do not reflect resveratrol bioavailability [57]. Resveratrol and its metabolites accumulate in tissue and organs due to the highly lipophilic affinity of resveratrol [58].

## 5. Conclusions

We have shown that 5-6 weeks of resveratrol treatment did not improve insulin sensitivity in DHT-induced PCOS rats, but exercise restored insulin sensitivity to a similar level as in control rats. Physical exercise also had beneficial effects on fat mass, adipocyte size, and estrus cyclicity. 

Although resveratrol did have positive effects on adiposity and cyclicity in a similar manner to exercise, resveratrol does not seem to be a good candidate for treating insulin resistance associated with PCOS because no improvement in insulin sensitivity was observed in PCOS rats on normal chow.

## Figures and Tables

**Table:** (a)

Gene	Forward primer 5′-3′	Reverse primer 5′-3′
*Cyp11a1 *	GCT GGA AGG TGT AGC TCA GG	CAC TGG TGT GGA ACA TCT GG
*Hsd3b1 *	CCA GAA ACC AAG GAG GAA T	CCA GAA ACC AAG GAG GAA T
*Cyp17a1 *	ACT GAG GGT ATC GTG GAT GC	CCG TCA GGC TGG AGA TAG AC
Hprt *	TTG TTG GAT ATG CCC TTG ACT	CCG CTG TCT TTT AGG CTT TG

**Table:** (b)

Gene	TaqMan assay ID	Gene	TaqMan assay ID
Hprt *	Rn01527840_m1	*Erra *	Rn00433142_m1
Ppia *	Rn00690933_m1	*Foxo1 *	Rn01494868_m1
Actb *	Rn00667869_m1	*Adipo *	Rn00595250_m1
Gapdh *	Rn01775763_g1	*Fndc5 *	Rn01519161_m1
*Ppargc1a *	Rn00580241_m1	*Pparg *	Rn00440945_m1
*Nrf1 *	Rn01455958_m1	*Sirt1 *	Rn01428093_m1

*Putative reference genes.

**Table 2:** Lipid profiles and fasting blood glucose and insulin levels measured after 5 weeks of resveratrol treatment or exercise.

(mmol/L)	Control-vehicle	Control-RSV	PCOS-vehicle	PCOS-RSV	PCOS-exercise
	(*n* = 10)	(*n* = 10)	(*n* = 10)	(*n* = 9)	(*n* = 9-10)
Cholesterol	2.44 ± 0.10	1.63 ± 0.26**	2.03 ± 0.09	2.05 ± 0.06	2.12 ± 0.14
Triglycerides	0.70 ± 0.11	0.51 ± 0.14	1.53 ± 0.20**	1.35 ± 0.23	1.22 ± 0.13
HDL	1.53 ± 0.10	0.88 ± 0.19*	1.48 ± 0.11	1.38 ± 0.10	1.51 ± 0.12
LDL	0.18 ± 0.03	0.15 ± 0.03	0.23 ± 0.03	0.32 ± 0.03	0.36 ± 0.05
Blood glucose	4.94 ± 0.15	5.33 ± 0.21	5.30 ± 0.08	4.93 ± 0.13	5.14 ± 0.20
Insulin (mU/L)	20.02 ± 2.99	17.81 ± 3.03	19.88 ± 1.68	18.65 ± 0.95	15.74 ± 2.05
HOMA-index	3.85 ± 0.31	3.52 ± 0.39	4.69 ± 0.42	4.19 ± 0.31	3.58 ± 0.47

Values are presented as mean ± SEM. Statistics were calculated using the Mann-Whitney *U* test. *P < 0.05, **P < 0.01 versus control-vehicle. HDL: high-density lipoprotein; LDL: low-density lipoprotein; HOMA: homeostasis model assessment; RSV: resveratrol.

**Table 3:** Effects of resveratrol and physical exercise on body composition measured by DEXA and bone mineral density measured by pQCT.

	Control-vehicle	Control-RSV	PCOS-vehicle	PCOS-RSV	PCOS-exercise
	(*n* = 9-10)	(*n* = 10)	(*n* = 9-10)	(*n* = 7–9)	(*n* = 9-10)
Fat gain (g)	16.3 ± 2.1	13.2 ± 1.3	24.7 ± 3.3*	15.3 ± 2.0^#^	1.1 ± 2.0^###^
Fat gain (% of BW)	1.9 ± 0.9	0.9 ± 0.4	2.5 ± 0.8	0.6 ± 0.5	−3.6 ± 0.5^###^
LBM gain (g)	61.2 ± 6.3	67.2 ± 4.5	86.0 ± 4.0**	79.6 ± 5.5	92.5 ± 7.6
LBM gain (% of BW)	−4.1 ± 0.8	−0.4 ± 2.1	−2.5 ± 0.7	−3.1 ± 1.5	2.6 ± 0.6^###^
BMC (% of BW)	2.75 ± 0.03	2.74 ± 0.05	2.57 ± 0.3***	2.54 ± 0.03	2.40 ± 0.04^##^
Tibia length (mm)	35.1 ± 0.3	35.2 ± 0.2	37.2 ± 0.2***	37.1 ± 0.4	36.8 ± 0.2
Total BMD (mg/cm^3^)	747 ± 14	760 ± 14	638 ± 19***	637 ± 17	643 ± 13
Trabecular BMD (mg/cm^3^)	480 ± 20	489 ± 23	313 ± 33*	294 ± 27	340 ± 22
Cortical BMD (mg/cm^3^)	1414 ± 3	1410 ± 5	1405 ± 3	1400 ± 5	1412 ± 3

Delta values before versus after treatment for fat and LBM are presented as mean ± SEM, and BMC and BMD are presented as after treatment values, mean ± SEM. Statistics were calculated using one-way ANOVA with Dunnett's *post hoc* test. *P < 0.05, **P < 0.01, ***P < 0.001 versus control-vehicle and ^#^ *P* < 0.05, ^##^ *P* < 0.01, ^###^ *P* < 0.001 versus PCOS-vehicle. BMC: bone mineral content; BMD: bone mineral density; LBM: lean body mass; RSV: resveratrol.

**Table 4:** Weight of dissected tissues after 5 weeks of resveratrol treatment or exercise.

	Control-vehicle	Control-RSV	PCOS-vehicle	PCOS-RSV	PCOS-exercise
	(*n* = 9)	(*n* = 10)	(*n* = 9)	(*n* = 9)	(*n* = 10)
Ovary (mg)	170 ± 7	171 ± 12	92 ± 15***	111 ± 19	127 ± 13
Uterus (mg)	639 ± 17	643 ± 41	257 ± 41**	389 ± 63	310 ± 46
Soleus (mg)	138 ± 11	147 ± 17	150 ± 12	146 ± 17	174 ± 6^#^
Soleus (g/kg BW)	0.48 ± 0.04	0.50 ± 0.05	0.43 ± 0.04	0.42 ± 0.04	0.48 ± 0.02^#^
Inguinal (g)	1.75 ± 0.20	1.53 ± 0.13	3.05 ± 0.24**	2.54 ± 0.22	1.74 ± 0.24^##^
Inguinal (g/kg BW)	5.9 ± 0.6	5.2 ± 0.4	8.7 ± 0.6**	7.4 ± 0.7	4.8 ± 0.6^###^
Parametrial (g)	5.84 ± 0.54	5.35 ± 0.45	7.85 ± 0.70	6.74 ± 0.65	5.33 ± 0.57^#^
Parametrial (g/kg BW)	19.8 ± 1.6	18.4 ± 1.5	22.2 ± 1.7	19.2 ± 1.5	14.7 ± 1.4^##^
Mesenteric (g)	3.11 ± 0.12	2.96 ± 0.14	2.74 ± 0.14	2.84 ± 0.18	2.66 ± 0.15
Mesenteric (g/kg BW)	10.5 ± 0.5	10.2 ± 0.4	7.8 ± 0.4**	8.1 ± 0.04	7.3 ± 0.4
Retroperitoneal (g)	2.76 ± 0.34	2.47 ± 0.21	3.02 ± 0.26	2.60 ± 0.28	1.82 ± 0.15^###^
Retroperitoneal (g/kg BW)	9.3 ± 1.1	8.5 ± 0.7	8.6 ± 0.7	7.4 ± 0.7	5.0 ± 0.4^###^

Values are presented as mean ± SEM values. Statistics were calculated with the Mann-Whitney *U* test. **P < 0.01, ***P < 0.001 versus control-vehicle and ^#^ *P* < 0.05, ^##^ *P* < 0.01, ^###^ *P* < 0.001 versus PCOS-vehicle. BW: body weight; RSV: resveratrol.

**Table 5:** Effects of resveratrol and physical exercise on steroidogenesis measured by RT-PCR.

Gene	Control-vehicle	PCOS-vehicle	PCOS-RSV	PCOS-exercise
	(*n* = 9)	(*n* = 9)	(*n* = 9)	(*n* = 9)
*Hsd3b1 *	1.00 ± 0.10	0.82 ± 0.09	0.84 ± 0.13	1.38 ± 0.14^##^
*CYP17a1 *	1.00 ± 0.57	5.09 ± 1.57*	4.82 ± 1.38	7.26 ± 2.434
*CYP11a1 *	1.00 ± 0.07	0.49 ± 0.11**	0.61 ± 0.18	0.78 ± 0.13

	Responders		PCOS RSV (*n* = 4)	PCOS exercise (*n* = 5)

*Hsd3b1 *			1.11 ± 0.19	1.63 ± 0.14^###^
*CYP17a1 *			2.71 ± 0.92	7.68 ± 4.31
*CYP11a1 *			1.04 ± 0.21^#^	1.01 ± 0.12^#^

	Nonresponders		PCOS RSV (*n* = 5)	PCOS exercise (*n* = 4)

*Hsd3b1 *			0.64 ± 0.14	1.11 ± 0.18
*CYP17a1 *			7.01 ± 2.04	7.65 ± 0.92
*CYP11a1 *			0.28 ± 0.17	0.53 ± 0.18

Values are presented as mean ± SEM of ΔΔCT values. Statistics were calculated using the Mann-Whitney *U* test. *P < 0.05, **P < 0.01 versus control-vehicle and ^#^ *P* < 0.05, ^##^ *P* < 0.01, ^###^ *P* < 0.001 versus PCOS-vehicle. RSV: resveratrol.
